# Protection of Rights of Knowledge Holders and Practitioners of Traditional Medicine in Tanzania

**DOI:** 10.24248/eahrj.v8i2.791

**Published:** 2024-06-26

**Authors:** Kijakazi Obed Mashoto

**Affiliations:** a National Institute for Medical Research

## Abstract

**Background:**

Lack or inadequate implementation and enforcement of legal frameworks for accessing and benefit sharing arising from the use of traditional and indigenous knowledge is associated with sub-optimal exploitation of traditional medicine knowledge and related research outputs in many African countries.

**Objectives:**

This study assessed the practice of protecting the rights of holders of traditional medicine knowledge, and identified challenges in regulating, registering and protecting traditional medicine based services, processes and products in Tanzania.

**Methods:**

Practice of protecting the rights of holders of traditional medicine knowledge in Tanzania was assessed through interviews with 12 knowledge holders and practitioners of traditional medicines, and 12 key informants from national regulatory authorities, and research and high learning institutions involved in traditional medicine research and development in Tanzania.

**Results:**

Absence of frameworks for accessing and benefit sharing arising from the use of tradition medicine knowledge, mistrust and inadequate knowledge on procedures for protection of traditional medicine based intellectual property hampers the practice of protecting the rights of holders of traditional medicine knowledge in Tanzania. Costly and bureaucratic procedures are among the challenges encountered by knowledge holders and practitioners of tradition medicine in registration of their traditional medicine products and practices. Poor network relationship between holders of traditional medicine knowledge and research community slow down the progress of traditional medicine research and development. Lack of guidelines for regulation of traditional medicine research may be the result of overlapping roles of the National Institute for Medical Research and the Council of Traditional and Alternative Medicine

**Conclusion:**

In Tanzania, the environment for protecting the rights of holders of traditional medicine knowledge is suboptimal. To stimulate exploitation of traditional medicine for economic growth, there is a need to develop and implement national and institutional frameworks for accessing and benefit sharing arising from the use of traditional knowledge.

## BACKGROUND

The Swakopmund Protocol on the Protection of Traditional Knowledge (TK) and Expression of Folklore defines the term traditional knowledge as any knowledge originating from a local or traditional community that is the result of intellectual activity and insight in a traditional context, including knowhow, skills, innovations, practices and learning, where the knowledge is embodied in the traditional lifestyle of a community, or contained in the codified knowledge systems passed on from one generation to another^1^. Traditional knowledge is not limited to a specific technical field, and may include agricultural, environmental or medical knowledge, and knowledge associated with genetic resources.^1^

The protection of Traditional Knowledge and Traditional Cultural Expressions (TCEs) as an autonomous Intellectual Property Rights (IPRs) is now becoming a reality around the globe and particularly in the African Continent. Currently negotiations on an international legal instrument for the protection of TK are taking place within the World Intellectual Property Organization (WIPO), particularly in the WIPO Intergovernmental Committee on Intellectual Property and Genetic Resources, Traditional Knowledge and Folklore, in order to address the positive protection of TK.^2^

The benefits derived from the legal protection of TK and TCEs necessitate the enactment of specific or incorporation of sufficient provisions within the existing legal framework. The international Intellectual Property Community has proposed for the adoption of the *sui generis* approach to the protection of TK and TCEs due to the challenges and inadequacy associated with the application of the existing Intellectual Property Rights. Some African countries have adopted the Swakopmund Protocol on the Protection of Traditional Knowledge and Expressions of Folklore.^1^ It is administered under the auspices of the African Regional Intellectual Property Organisation (ARIPO). The Swakopmund Protocol on the Protection of Traditional Knowledge and Expressions of Folklore within the Framework of the African Regional Intellectual Property Organization (ARIPO)^1^ was adopted on August 9, 2010, amended on December 6, 2016 and, revised and reprinted in 2019.^1^ The Protocol so far has been signed by ten ARIPO members, namely: Botswana, Ghana, Kenya, Lesotho, Liberia, Mozambique, Rwanda, Namibia, Zambia and Zimbabwe. These countries have subsequently enacted local legislations in order to meet their obligations under the Protocol. Kenya, for instance, adopted the Traditional Knowledge and Cultural Expressions Act in 2016. Tanzania have not adopted the protocol, and hence risking Traditional Knowledge and Traditional Cultural Expressions to be exploited by third parties without consultation of, and benefit sharing with, the community from which the knowledge or expressions originate.

The current World Health Organization (WHO) Traditional Medicine Strategy^3^ build upon the framework for action laid out in the first WHO Traditional Medicine Strategy,^4^ the traditional medicine sections of the 2004 – 2007 WHO Medicines Strategy^5^ and the traditional medicine components of the 2008 – 2013 WHO Medicines Strategy.^6^ It aims to support Member States in developing proactive policies and implementing action plans that will strengthen the role of traditional medicine (TM) in keeping populations healthy. The objectives of the strategy include 1) promote integration of TM within national health care systems, where feasible, by developing and implementing national TM policies and programmes, 2) promote the safety, efficacy and quality of TM by expanding the knowledge base, and providing guidance on regulatory and quality assurance standards, 3) increase the availability and affordability of TM, with an emphasis on access for poor populations and 4) promote therapeutically sound use of appropriate TM by practitioners and consumers.

Over the years, WHO regional office for Africa spearheaded the implementation of a regional strategy^2^ endorsed by African Heads of State in Lusaka, Zambia to promote the role of traditional, complementary and alternative medicine in health systems in the African region. Apart from short and long term traditional medicine professional courses, and training of traditional medicine practitioners and holders of traditional knowledge, the regional plan also promoted local production and cultivation of medicinal plants, as well as the establishment of intellectual property rights for traditional medicine knowledge in few nations.^2^

Despite significant progress made in implementing previous strategies and frameworks for traditional medicine around the world, Member States continue to experience challenges related to development and enforcement of policy and regulations; integrating TM into national and primary health care (PHC); regulation of safety and quality traditional medicine practice of practitioners, evaluation of efficacy of TM products; ability to control and regulate traditional and complementary medicine (T&CM), research and development, education and training of T&CM practitioners, and sharing information about policies, regulations, service profiles and research data, or obtaining reliable objective information resources for consumers.^7-9^

In Tanzania, the existence of traditional medicine practitioners and protection of their rights to practice is well recognized to the extent that Traditional Medicine Research Unit at the University of Dar es Salaam was established in 1974, and later elevated to an Institute of Traditional Medicine (ITM) in 1991. In 1989, a unit of traditional medicine was established within the ministry of health. In response to the WHO African Regional Strategy to promote traditional medicine,^3^ the Act which established the Council of Traditional and Alternative Medicine was passed in 2002.^10^ In the same year, the National Institute for Medical Research (NIMR) which is responsible for conducting, coordinating, promoting and regulating traditional medicine research in Tanzania, established the department of traditional medicine research. In 2021, this department was promoted to a centre, which is known as NIMR Mabibo Traditional Medicine Research Centre. The functions of this centre are two folds: 1) conduct traditional medicine research and 2) massive production and commercialization of efficacious traditional medicines.

At the Sokoine University of Agriculture (SUA) and the University of Dar es Salaam (UDSM), the departments of Botany, Chemistry, Marine Sciences and Microbiology are all routinely involved in teaching as well as researching in traditional medicine and natural products. Tanzania Medicines & Medical Devices Authority is charged with the responsibility of regulating and controlling medicines, medical devices, herbal drugs and diagnostics. Furthermore, the government have prioritized traditional medicine research in its HSSP V and aims to enable the availability of safe and sustainable alternative treatment offered in conjunction with formal health care.^11^

In realization of potential of high learning and research institutions, and holders of traditional knowledge to contribute to the economic growth, the Ministry of Education, Science and Technology developed guideline which explicitly describes the role of COSTECH in supporting holders of traditional medicine knowledge and research community to protect, commercialize or add value to the identified innovations, inventions and traditional knowledge practice and products.^12^ While the Council of Traditional and Alternative Medicine^10^ register traditional medicine practices and products, COSTECH, provides support to holders of TK to protect and commercialize innovations and inventions arising from the use of traditional knowledge.^12^ However, the country does not have legislations or specific frameworks for legal protection of TK.^13^ The main objective of this study was to assess the practice of protecting the rights of holders of traditional medicine knowledge, and identify challenges in regulating, registering and protecting traditional medicine based services, processes and products in Tanzania.

## METHODS

### Study Design and Participants

A qualitative, descriptive, and exploratory study design was employed to assess practice of research institutions, universities, and quality assurance and regulatory institutions in protecting the rights of holders of traditional medicine knowledge, and challenges associated with protection of traditional medicine based intellectual property in Tanzania.

In total 24 key informants were purposeful selected from research and high learning institutions involved in traditional medicine research and development, regulatory authorities of traditional medicine, and traditional medicine association (Table 1). Through the registrar of the Traditional and Alternative Medicine Council, database of the registered practitioners of traditional medicine and owners of the registered traditional medicine or herbal products was accessed. The database of practitioners of the traditional medicine was stratified by sex, and from each stratum, 3 key informants were randomly selected. Out of 6 sampled practitioners of traditional medicine, three were residents of Morogoro, Singida, and Dodoma Regions respectively. The sampled practitioners of traditional medicines were contacted, and each was requested to provide three names of holders of traditional medicine knowledge in his/her community. From the list provided by each practitioner of traditional medicine, one knowledge holder was randomly sampled.

**Table 1.** Number of Key Informants

Key informant	Number
National Institute for Medical Research (NIMR)	2
Commission of Science and Technology (COSTECH)	2
University of Dar es Salaam (UDSM)	2
Institute of Tradition Medicine (ITM)	1
Ministry of Health – Traditional Medicine Unit (MOH-TM Unit)	1
Council of Traditional and Alternative Medicine	1
Sokoine University of Agriculture (SUA)	1
Tanzania Medicine and Medical Devices Authority (TMDA)	1
Government Chemist Laboratory Agency (GCLA)	1
Female knowledge holders of traditional medicine	3
Male knowledge holders of traditional medicine	3
Female practitioners of traditional medicine	3
Male practitioners of traditional medicine	3
Total	24

Knowledge holders of traditional medicine included individuals who knows a particular plant which treat a certain disease. Knowledge owner may not necessarily practice traditional medicine. Individuals who practice traditional medicine are known as practitioners of traditional medicine or traditional healers.^10^

### Data Collection

Two in-depth interview guides were used to conduct interviews, one was for holders and practitioners of traditional medicine, and the other one was for key informants from research and high learning institutions involved in traditional medicine research and development, and regulatory of traditional medicine in the country. The tools were designed to capture information on respect to and the rights of knowledge holders and practitioners of TM, network relationship between knowledge holders and practitioners of TM, and institutions responsible for TM research and regulation, existence and implementation of frameworks for accessing and benefit sharing following the use of traditional medicine knowledge (TMK). Face to face interviews were conducted using Kiswahili language. However, 10 key informants residing outside Dar es Salaam were subjected to telephone interviews, and they included key informants from TMDA, Tanzania Traditional and Alternative Medicine Council, Ministry of Health – Traditional Medicine Unit, SUA, 3 practitioners of traditional medicine residents of Morogoro, Singida, and Dodoma, 3 holders of traditional knowledge residents of Morogoro, Singida, and Dodoma respectively.

All interviews were audio recorded and then transcribed and translated into English. In addition, mandates and functions of institutions which are involved in regulation of TM practices and products, and conduction and regulation of TM research and development (R&D) were reviewed. Thus, the mandates and functions of Tanzania Commission of Science and Technology (COSTECH), Council of Traditional and Alternative Medicine, the National Institute for Medical Research (NIMR), Institute of Traditional Medicine (ITM), Tanzania Medicines and Medical Devices Authority (TMDA), Government Chemist Laboratory Agency (GCLA), Muhimbili University of Health and Allied Sciences (MUHAS), Sokoine University of Agriculture (SUA) and University of Dar es Salaam (DSM) were reviewed.

### Data Analysis

Audios were first transcribed by two research assistants. Translation of the transcribed audios was done by the principal investigator of the study. A stepwise approach was used for a deductive thematic analysis of the interview transcripts. First, research questions were examined to generate a coding framework with several themes. Individual transcripts and codes representing participants' responses to the questions were exported to relevant themes and related key issues (Table 2) within the coding framework. The codes were excluded when they did not provide critical value to the study. The coded data were exported to Microsoft Word (Microsoft Corporation) for interpretative analysis and report generation.

**Table 2.** Themes and Key Issues

Themes	Key issues
Regulation of traditional medicine practice and research in Tanzania	Overlapping roles Inadequate understanding of different institutions' roles in regulation of tradition medicine practice and research
Registration and protection of traditional medicine based intellectual property and rights of knowledge holders	Inadequate IP awareness and knowledge among rights holders Lack of frameworks for protection of traditional medicine knowledge Lack of mechanisms for benefit sharing arising from the use of traditional medicine knowledge Bureaucratic procedures and high costs Network relationships between holders of traditional medicine knowledge and research community Mistrust Unclear working, collaboration or partnership agreements

### Ethical Considerations

Waiver for ethical approval to conduct the interviews was granted by the Medical Research Coordinating Committee (Ref number NIMR/HQ/R.8a/Vol II of 2022/122). Study participants signed informed consent prior to interview.

## RESULTS

### Overlapping Roles of Different Institutions in Regulation of Traditional Medicine Practice and Research

Council of Traditional and Alternative Medicine was established by Act^10^ number 23 of 2002 and mandated to monitor, regulate, promote, and support the research development of traditional medicine. Subject to the provisions of the Patents Act, 1987, the Minister of Health through the Council of Traditional and Alternative Medicine may provide for matters related to patenting of traditional medicine inventions. In the performance of its functions, the Council of Traditional and Alternative Medicine maintains a system of consultation with NIMR, ITM, TMDA, GCLA and, Chemistry and Botany Departments of University of Dar es Salaam. The Council make decisions on registration of the submitted herbal or traditional medicine applications based on quality assurance assessment reports from GCLA and Chemistry Department of UDSM, and report of *materia medica* used in herbal medicine from Botany Departments of UDSM.

Although the Council of Traditional and Alternative Medicine have guidelines in place for registration of traditional and alternative medicine practice and products, there are no guidelines for regulation of traditional medicine research. According to the Act which established the Council of Traditional and Alternative Medicine, R&D Committee of the Council of Traditional and Alternative Medicine is responsible for developing guidelines for regulation of traditional medicine research and setting standards for conduction of traditional and alternative medicines research. Review of key documents and interviews with key informants revealed no clear linkage between the R&D Committee of the Council of Traditional and Alternative Medicine and national and institutional research ethics committees. It should be noted that, apart from conducting traditional medicine research, the National Institute for Medical Research is also responsible for promoting and regulating the conduction of traditional medicine in Tanzania. Other institutions, which also conduct traditional medicine research, include ITM, SUA, UDSM and Ifakara health Institute.

Whereas the Council of Traditional and Alternative Medicine is responsible for registration and monitoring of provision of traditional medicine services and use of safe herbal or traditional medicine products, TMDA provide approval for herbal or traditional medicine products whose clinical trial results indicate that the herbal product is safe and efficacious in treating or preventing a certain disease or condition. Interviews with holders of traditional medicine knowledge clearly indicated their limited awareness and knowledge regarding the roles of different public institutions in promoting, coordinating and regulating traditional medicine practice, products and research. Majority of holders of traditional medicine knowledge thought or believed that NIMR, ITM, UDSM and GCLA are responsible for approving the use of herbal medicine.

*“I am longing to be granted intellectual property rights of my herbal medicines because there are people who are using my name and the names of my products. Unfortunately, there is nothing I can do about it because as we speak my traditional medicine products have not been registered with the Council of Traditional and Alternative Medicine. Once they are registered [her herbal products] I will start the process of getting them protected, and you [NIMR] should tell me how much will it cost and where should I go to apply for intellectual property rights”*

(Respondent-9 Knowledge Holders and Practitioners of Traditional Medicine)

### Registration and protection of traditional medicine based intellectual property and rights of knowledge holders

Almost all the interviewed holders of traditional medicine knowledge said that they needed to register their herbal medicine not because of the law requirements but they also believed that registered herbal medicines are more trusted than those with no registration, and it is easier to penetrate the market when herbal medicine is registered. However, bureaucratic procedures and high costs of registration prevent holders of traditional medicine knowledge and practitioners from registering their products and services respectively.

*“When holder of traditional medicine knowledge wants to register the product, we provide him/her with our [Council] form specifying the parameters to be measured and direct to go to the department of Botany for plant identification, and from there he/she go to either GCLA or Chemistry Department of UDSM for quality assurance assessment of his/her herbal product. We [Council] use assessment report from GCLA and/or Chemistry Department of UDSM to make registration decision. The biggest outcry from traditional medicine practitioners is high costs associated with the registration of herbal medicine”*

(Respondent 5-Rsearch, Regulatory and Quality Assurance Institutions).

*“It took a year to get the registration certificate from Council of Traditional and Alternative Medicine. By the time I received it, it was already the time for renewal*

(Respondent 1-Knowledge Holders and Practitioners of Traditional Medicine)

*“Because of bureaucracy in this country, I registered my herbal medicine in Kenya. Had it not been for bureaucracy we would have been very far by now. I am registered with the Council and my facility is registered, but I cannot register my herbal medicines here because it is too expensive, and registration procedures are very bureaucratic. Too much back and forth movements, to GCAL, NIMR and the UDSM before you go back to the Council”*

(Respondent 3-Knowledge Holders and Practitioners of Traditional Medicine).

*“At GCLA office I was told that quality assurance assessment of one herbal medicine will cost USD 281, so if I have many herbal medicines I will not be able to afford the cost. Most traditional medicine practitioners in rural areas may not be able to register their herbal medicine”*

(Respondent 8-Knowledge Holders and Practitioners of Traditional Medicine.

Although all key informants knew the importance of intellectual property rights, limited knowledge on what should be protected and what steps to be taken to protect intellectual property created through innovation in traditional medicine impacted negatively on the use of intellectual property system. Only two out of 12 (about 17%) of the interviewed traditional medicine practitioners reported to have used intellectual property system.

*I submitted my application to BRELA three years ago but until now I have not been granted any intellectual property rights. Therefore, I have decided to name my herbal products using traditional names from my tribe, to make it hard for others to use the names of which they do not understand meanings*

(Respondent 3-Knowledge Holders and Practitioners of Traditional Medicine).

*Yes, it is a year now since I registered my products. I have 15 products, and 5 of them have BRELA registration and barcodes. No other person can use my products' names and tradename without my consent*

(Respondent 1-Knowledge Holders and Practitioners of Traditional Medicine).

*“I have submitted my application to BRELA for to registration of my herbal medicines but to date I have not received any response. Yeah, there are many advantages of using intellectual property system, but the costs may not be affordable by most of holders of traditional medicine knowledge”*

(Respondent 12-Knowledge Holders and Practitioners of Traditional Medicine)

*“Intellectual property system? No, I have never used it. You know we do not earn much from selling traditional medicine products. It is really a struggle to prepare traditional medicine products, and hence it pains when another person from nowhere imitates what you have prepared, and very often the imitation does not meet standards of the original products. In this way, you stand a good chance of losing customers”*

(Respondent 5-Knowledge Holders and Practitioners of Traditional Medicine)

While key informants from different public institutions said that the government recognize and respects the contribution of traditional medicine in improving health and wellbeing of its citizens, majority of traditional medicine practitioners had different views with regard to respect for traditional medicine.

*“We are part of the community which believe that traditional medicine can be used to treat various diseases. The fact that we have a practice of reaching out to traditional medicine practitioners and ask them what type and parts of plants do they use to treat certain diseases, shows that we respect traditional medicine. We use the collected information to conduct scientific research to prove their claims”*

(Respondent 2-Research, Regulatory and Quality Assurance Institutions).

*“People in general have little respect for traditional medicine that is why even the price of herbs in the market is very low”*

(Respondent 10-Knowledge Holders and Practitioners of Traditional Medicine).

*“We have been neglected for so long until recently when the government wanted traditional medicine practitioners to come up with herbal medicine for COVID 19”*

(Respondent 3-Knowledge Holders and Practitioners of Traditional Medicine).

### Linkage Between Holders of Traditional Medicine Knowledge and Research Community

There is weak link between holders of traditional medicine knowledge and public institutions responsible for coordination, promotion and regulation of traditional medicine practice and research. Mistrust and lack of or unclear mechanisms for collaborating in tradition medicine research are the main reasons for the weak linkage.

*“It took me more than a year to convince one of the institution that I have something that is effective in treating HIV. Unfortunately, our collaboration in researching the efficacy and safety of my traditional medicine to treat HIV ended before it started. The institutions in this country are being extractive, all they want is to get the knowledge we have”*

(Respondent 4-Knowledge Holders and Practitioners of Traditional Medicine).

Some of the holders of traditional medicine knowledge have inadequate understanding of what is research and believed that traditional medicine research is not important as illustrated in the quote below.

*There is no need to re-research, because we [traditional medicine practitioners] conduct research before we *seek* registration for our herbal medicines. I do not depend on anyone to conduct research, I do it myself. The job of universities and research institutions is documentation and not going to the forests to look for medicinal plants. Government should make use of us; we are capable of treating diseases with herbal medicines”*

(Respondent 5-Knowledge Holders and Practitioners of of Traditional Medicine).

Other holders of traditional medicine knowledge felt that they are being side-lined and not respected by the institutions which are mandated to coordinate, regulate and promote traditional medicine practices and research.

*We [holders of traditional medicine knowledge] get no support from the Council of Traditional and Alternative Medicine, because they also have herbal medicines. As a matter of fact, many government officials claimed to have developed herbal medicine when Corona was at its peak, we don't know where they were before Corona”*

(Respondent 1-Knowledge Holders and Practitioners of Traditional Medicine)

*“We are being discriminated by institutions, they disrespect us [traditional medicine practitioners] and they [research institutions and universities] do not know that we are the pharmacists of traditional medicine. We mix medicinal plants based on seasonality. I am wondering now how come the institutions are doing what we are doing, preparing and selling herbal medicines, it is not their job. They [institutions] do not want to work with us; we do not have good relationship with them. I went to one institution to submit my herbal products but I was told that they would call me back because they were busy with other government duties. I felt disrespected and our conversation did not go well”*

(Respondent 2-Knowledge Holders and Practitioners of Traditional Medicine).

Few of holders of traditional medicine knowledge who ever worked with research community did not want to disclose their knowledge. Others were sceptical to use intellectual property system for fear of exposing their traditional medicine secrets.

*“The problem is, one has to go to various offices such as department of botany and chemistry at UDSM, TMDA, TBS and GCLA for quality assurance assessment before one's herbal or traditional medicinal product is registered by the Council. My worry is who will ensure that such institutions do not use the submitted information [herbal medicine] for their own gains. Because both research and quality assurance institutions are blamed for stealing traditional medicine practitioners' knowledge”*

(Respondent 4-Research, Regulatory and Quality Assurance Institutions).

*“Why would I want to apply for intellectual property rights for my herbal medicine? Research institutions, the Council and BRELA use the same extractive process. I cannot expose the secret of my herbal medicine. Honestly, I don't think this is right. Why they [BRELA, research institutions, universities and Council of Traditional and Alternative Medicine] want holders of traditional medicine knowledge to disclose? I am telling you, if you see the forms, you will be shocked. First of all, in this country we do not have frameworks for protecting rights of holders of traditional medicine knowledge, so we don't want to disclose without being sure that our rights will be protected. Let us not deceive ourselves; the country does not have IPR system for holders of traditional medicine knowledge”*

(Respondent 4-Knowledge Holders and Practitioners of Traditional Medicine)

*“What I have come to realize is that those with responsibility of regulating and promoting traditional medicine take advantage of their positions by using our knowledge for their personal gains. I had submitted my herbal medicine for research to one of the public institutions, but when I raised the issue of contract, the doctor I was supposed to work with suddenly disengaged. Many holders of traditional medicine knowledge are worried that their knowledge will land into bad hands”*

(Respondent 1-Knowledge Holders and Practitioners of Traditional Medicine)

*“Based on their institution IP policy I knew my share of benefit would be 50%, but I did not understand why the institute was forcing me to disclose. Our contractual agreement required me to supply herbal medicine and that the institute will be responsible for research component. I don't think there is any institution which have pure intention to promote us [holders of traditional medicine knowledge]; they just want take our knowledge and claim ownership. They told me to submit all my materia medica and disclose the formula and everything. I am glad that I did not comply; I would have been the looser. I said no to submission of materia medica and said no to disclosure”*

(Respondent 4-Knowledge Holders and Practitioners of Traditional Medicine).

*“As an institute we have developed contractual agreement which provides for mechanisms for partnership and collaboration, or consultancy. It is unfortunate that most holders of traditional medicine knowledge, who come to us with the intention of researching their herbal medicines, are not happy with one clause which requires them to disclose. We have production agreement and research contractual agreement which do not provide for mechanisms of benefit sharing. Some holders of traditional medicine knowledge do not want to disclose because they know what they have is not something new, but they use different names for the same products”*

(Respondent 1-Research, Regulatory and Quality Assurance Institutions).

Lack or sub-optimal implementation of policies for networking relationship, working or collaboration contract, and lack of knowledge on procedures for IP protection, and lack of frameworks for accessing and sharing benefits arising from the use of traditional medicine knowledge may have contributed to the observed tendency of holders of traditional medicine knowledge not to trust research and regulatory communities.

*“Major challenge is mistrust. Holders of traditional medicine knowledge do not trust us [researchers]; they think we want to use their knowledge for our personal gains. Nevertheless, it is a pity that they have neither capacity nor resources to protect their traditional medicine innovation and technology. Something needs to be done to address this challenge which impacts the development of traditional medicine in this country”*

(Respondent 3-Research, Regulatory and Quality Assurance Institutions).

## DISCUSSION

In line with findings of a recently published study,^14^ holders and practitioners of traditional medicine recognize that the Council of Traditional and Alternative Medicine is the regulatory body for traditional medicine practice and products. However, majority of interviewed holders of traditional medicine knowledge demonstrated low awareness regarding the different regulatory, coordinating and promoting roles of various public institutions such as NIMR, ITM, UDSM, TMDA, BRELA and GCLA.

Research and development institutions in Tanzania have inadequate capacity to effectively engage in commercial or entrepreneurial activities; and the holders of traditional knowledge do not have capital resources for R&D and commercialization of their products or practices either.^12,13^ Despite the existence of the guideline document which describes the role of COSTECH in supporting knowledge holders of traditional medicine and research community,^12^ none of the study participants was aware of such document. It is possible that the guideline document was not widely disseminated and not packaged in a user friendly format for different audiences. Low awareness on COSTECH's role in promoting protection and commercialization of traditional knowledge based IP is further complicated by limited knowledge and low use of IP system among traditional knowledge holders and research community.^13^

Information on traditional medicine practice and safety of the traditional medicine products is critical to guide the public because many people in developing countries access traditional medicine when in need of healthcare before accessing contemporary medicine or in parallel.^15-20^ In Tanzania, nearly 70% of people frequently access healthcare through traditional healers or vendors.^21,22^ Of those, 15% of users of traditional medicines have chronic diseases, and many of them have poor biological understanding of those chronic diseases.^21^ The practice of traditional medicine is also accompanied with malpractice that puts the health of its clients at risk.^23,24^ Hence limited access to traditional medicine information or the presence of imperfect information among practitioners and regulators can easily cause non-adherence to and poor implementation of regulations, respectively.^9^ It is therefore important to revisit some of the R&D Committee of the Council of Traditional and Alternative Medicine^10^ and ensure that both practitioners and regulators have access to evidence based information generated by high quality traditional medicine research. Apart from improving efficiency in registering practices and products of traditional medicine, establishing a sub-committee of Traditional Medicine Research Review sub-committee of the National Health Research Review Committee may help to effectively regulate the conduction of traditional medicine research in the country.

According to the Tanzania Traditional Medicine Act,^10^ traditional and alternative medicine practice and products must be licensed. Our results revealed that holders and practitioners were not satisfied with the registration and licensing process. All interviewed knowledge holders and practitioners of traditional medicine complained of the high costs, and cumbersome and bureaucratic registration processes. Similar to the findings of another study, practitioners criticized the procedure and termed it as a barrier to development of the traditional medicine sub-sector in the country.^14^

The belief that registered herbal medicines are more trusted, and can be easily commercialized is in line with the findings of the study, which was conducted in Moshi, Tanzania, whereby traditional healers and herbal vendors expressed the importance that credibility plays in their business.^17^

The challenges reported in this study ranged from mistrust, limited knowledge on registration and protection procedures to poor network relationships between traditional knowledge holders, research community and regulators of practice and products of traditional medicine.

In Tanzania, there is neither a comprehensive legislation nor incorporation of some provisions, in any of the existing legislation, which offers protection of traditional knowledge. The protection of Traditional Knowledge and Traditional Cultural Expressions is essential for the benefit of the holders as well as for national development. Knowledge holders and other stakeholders of traditional medicine in Tanzania hardly use the existing IP system for protection of TK. The country should think of adopting sui generis approach for effective protection of TK.

Mistrust which lead to fear of disclosure, and poor network relationship between TK holders, regulators and research community impact negatively on the development of traditional medicine in the country. This challenge can partly be addressed by developing local or adopting and implementing international or regional frameworks for accessing and sharing the benefits arising from the use of traditional knowledge.

### Strengths and Limitations of the Study

This study triangulated information from various sources such as research and academic institutions, regulatory bodies, holders of TMK and practitioners of TM. However, half of the interviewed practitioners of traditional medicine and holders of knowledge were from Dar es Salaam, and the remaining half were from Morogoro, Singida, and Dodoma. Due to cultural diversity and differences in social norms, the interviewed TMK holders and TM practitioners represented the Eastern and Central Zones of the country. Thus, their views may differ with the views of these from other five Zones of the country. In future, researchers should consider to include representatives from each region or Zone.

## CONCLUSIONS

In the absence of formal mechanisms for accessing and sharing benefits arising from the use of traditional medicine knowledge, it will be difficult for regulators and research community to establish good network relationship with TK holders and to effectively protect their rights. To improve efficiency in registration of traditional medicine practice and products, the government should think of establishing one-stop centre. Social behavioral communication change interventions for TK holders and research community should be developed and implemented in order to increase use of IP system. Short and long term IP training may help to close IP knowledge gap among traditional knowledge holders and research community. Furthermore, an examination on how investments in social and behavioral change interventions for researchers, regulators, knowledge holders and practitioners is needed to find out how such investments might reduce mistrust and improve the registration efficiency, and increase use of IP system by research community and holders of traditional knowledge, and increase commercialization of traditional knowledge based innovations in Tanzania.
